# FGFR3–TACC3 cancer gene fusions cause mitotic defects by removal of endogenous TACC3 from the mitotic spindle

**DOI:** 10.1098/rsob.170080

**Published:** 2017-08-30

**Authors:** Sourav Sarkar, Ellis L. Ryan, Stephen J. Royle

**Affiliations:** Centre for Mechanochemical Cell Biology, Warwick Medical School, University of Warwick, Gibbet Hill Road, Coventry, CV4 7AL, UK

**Keywords:** mitosis, FGFR3–TACC3, TACC3, spindle, bladder cancer

## Abstract

Fibroblast growth factor receptor 3–transforming acidic coiled-coil containing protein 3 (FGFR3–TACC3; FT3) is a gene fusion resulting from rearrangement of chromosome 4 that has been identified in many cancers including those of the urinary bladder. Altered FGFR3 signalling in FT3-positive cells is thought to contribute to cancer progression. However, potential changes in TACC3 function in these cells have not been explored. TACC3 is a mitotic spindle protein required for accurate chromosome segregation. Errors in segregation lead to aneuploidy, which can contribute to cancer progression. Here we show that FT3-positive bladder cancer cells have lower levels of endogenous TACC3 on the mitotic spindle, and that this is sufficient to cause mitotic defects. FT3 is not localized to the mitotic spindle, and by virtue of its TACC domain, recruits endogenous TACC3 away from the spindle. Knockdown of the fusion gene or low-level overexpression of TACC3 partially rescues the chromosome segregation defects in FT3-positive bladder cancer cells. This function of FT3 is specific to TACC3 as inhibition of FGFR3 signalling does not rescue the TACC3 level on the spindle in these cancer cells. Models of FT3-mediated carcinogenesis should, therefore, include altered mitotic functions of TACC3 as well as altered FGFR3 signalling.

## 1. Introduction

Gene fusions are the result of structural chromosomal rearrangements and are considered driver mutations in cancer [1]. Besides classical fusions detected in blood cancers, fusion genes are also found in several solid tumour types including bladder carcinoma [2]. Bladder cancer is mainly associated with an activating point mutation in fibroblast growth factor receptor 3 (FGFR3) which has been found in more than 80% of cases of low-grade tumours; however, an FGF3–transforming acidic coiled-coil containing protein 3 (FGFR3–TACC3; FT3) fusion gene has recently been identified in bladder cancer [3].

FT3 is formed due to intrachromosomal rearrangement of chromosome 4, between FGFR3 and TACC3. The fusion has been identified in other cancers including glioblastoma multiforme and non-small cell lung cancer [4]. FGFR3 belongs to the FGFR family of receptor tyrosine kinases [5]. Normally, FGFR is activated by FGF-heparin binding to monomeric FGFRs, resulting in dimerization and transphosphorylation in the cytoplasmic tyrosine kinase domain. This event leads to activation of several downstream signalling pathways including MAPK [6]. Aberrant activation of FGFR signalling has been implicated in cell proliferation and tumourigenesis [6].

TACC3 is a cancer-associated protein belonging to the TACC family [7], and is important for mitotic spindle stability [8,9]. Aurora-A-mediated phosphorylation of TACC3 is important for its association with clathrin and localization to mitotic spindle microtubules [10–12]. TACC3, together with clathrin and ch-TOG, forms a ternary complex which acts as a cross-linker between adjacent microtubules (MTs) [10,13]. Recently, it has been shown that such cross-links make a network, termed ‘the mesh’ which is required for organization of kinetochore fibre MTs [8]. Moreover, it has been demonstrated that alteration of TACC3 levels causes disorganization of kinetochore fibres and alters mitotic progression and normal chromosome segregation [8,14,15]. TACC3 levels are found to be altered in many human cancers including bladder cancer [16]. Either an increase or a decrease in TACC3 levels results in a mitotic delay together with mitotic defects [8,9,17]. Such defects during mitosis can lead to aneuploidy and may contribute to cancer initiation and/or progression [18]. TACC3 contains a C-terminal coiled-coil domain (TACC domain, 638–838 aa) which enables dimerization of TACC3 and even its multimerization under certain circumstances [7,19,20]. Since the TACC domain features in the FT3 fusion, it is thought that this results in constitutive activation of FGFR3 signalling, similar to the mechanism of other fusions involving receptor tyrosine kinases [21,22].

The investigation of the role of FT3 in oncogenesis has so far focused on the role of FGFR3 signalling, while the impact on TACC3 has been relatively unexplored. It was suggested that the presence of a TACC3 domain in FT3 results in a spindle pole localization during mitosis and thereby causes severe mitotic defects and aneuploidy [22]. Inhibition of FGFR3 kinase activity by PD173074 rescued FT3-induced aneuploidy. However, the exact mechanism by which FT3 induces such defects was not addressed. Here, we investigate the association between FT3 and mitotic defects observed in bladder cancer cells and describe a TACC3-specific role for FT3 in this process.

## 2. Results

### 2.1. FT3 causes mitotic defects but does not localize to the mitotic spindle

**Figure 1.**
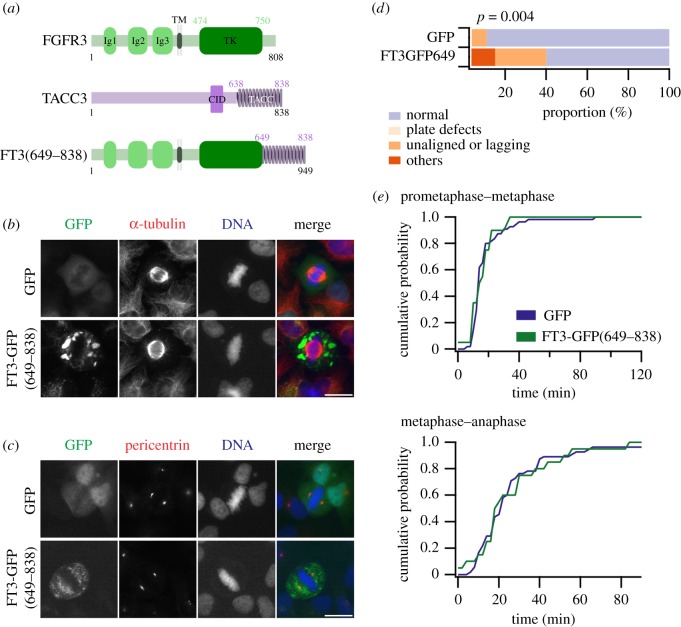
FGFR3–TACC3 fusion proteins (FT3) do not localize to the mitotic spindle but cause mitotic defects. (*a*) Schematic diagrams of FGFR3, TACC3, and a fusion of FGFR3 and TACC3, FT3(649–838). (*b*,*c*) Representative images of HeLa cells expressing GFP or FT3-GFP(649–838) and stained for α-tubulin (*b*) or pericentrin (*c*). Scale bar, 10 µm. (*d*,*e*) Live-cell imaging of HeLa cells co-expressing H2B-mCherry with either GFP or FT3-GFP(649–838) to monitor mitotic progression. (*d*) Quantification of cells with normal mitosis and cells that display various abnormal phenotypes (metaphase plate defects, unaligned or lagging chromosomes or other segregation defects). (*e*) Cumulative histograms of mitotic progression of all cells. Time from nuclear envelope breakdown to final chromosome alignment (prometaphase–metaphase) and from metaphase to beginning chromosome segregation (metaphase–anaphase) is shown for the conditions indicated. The full mitotic progression dataset is shown in the electronic supplementary material, figure S1.

It was previously shown that glioblastoma cells with FT3 gene fusions have defects in chromosome segregation [22]. This was attributed to FT3 localizing to the spindle pole and somehow affecting normal mitosis. We began investigating the role of FT3 in chromosome segregation by examining the localization of FT3-GFP in HeLa cells. FT3-GFP(649–838) did not localize to the mitotic spindle nor to centrosomes but was present outside the spindle region in structures reminiscent of membrane vesicles (figure 1*b*,*c*). The presence of FT3 in vesicles is to be expected for a transmembrane protein. This result is similar to the previous report in glioblastoma cells [22].

Next, to see the effect of FT3 on mitosis, live-cell imaging was carried out in asynchronously growing HeLa cells expressing FT3-GFP(649–838) or GFP as a control, together with H2B-mCherry to visualize chromosome dynamics. Expression of FT3-GFP(649–838) increased the number of abnormal mitotic figures compared with expression of GFP (figure 1*d*). Expression of this fusion did not cause a delay in mitotic progression (nuclear envelope breakdown to anaphase) compared with control cells expressing GFP (figure 1*e*). Again, this is similar to a previous report using Rat1A cells [22]. These results suggest that expression of FT3 induces defects in mitosis, but that this effect is independent of localization at the spindle pole or mitotic spindle.

### 2.2. FT3 induces mitotic defects by altering the levels of endogenous TACC3 at the mitotic spindle

**Figure 2.**
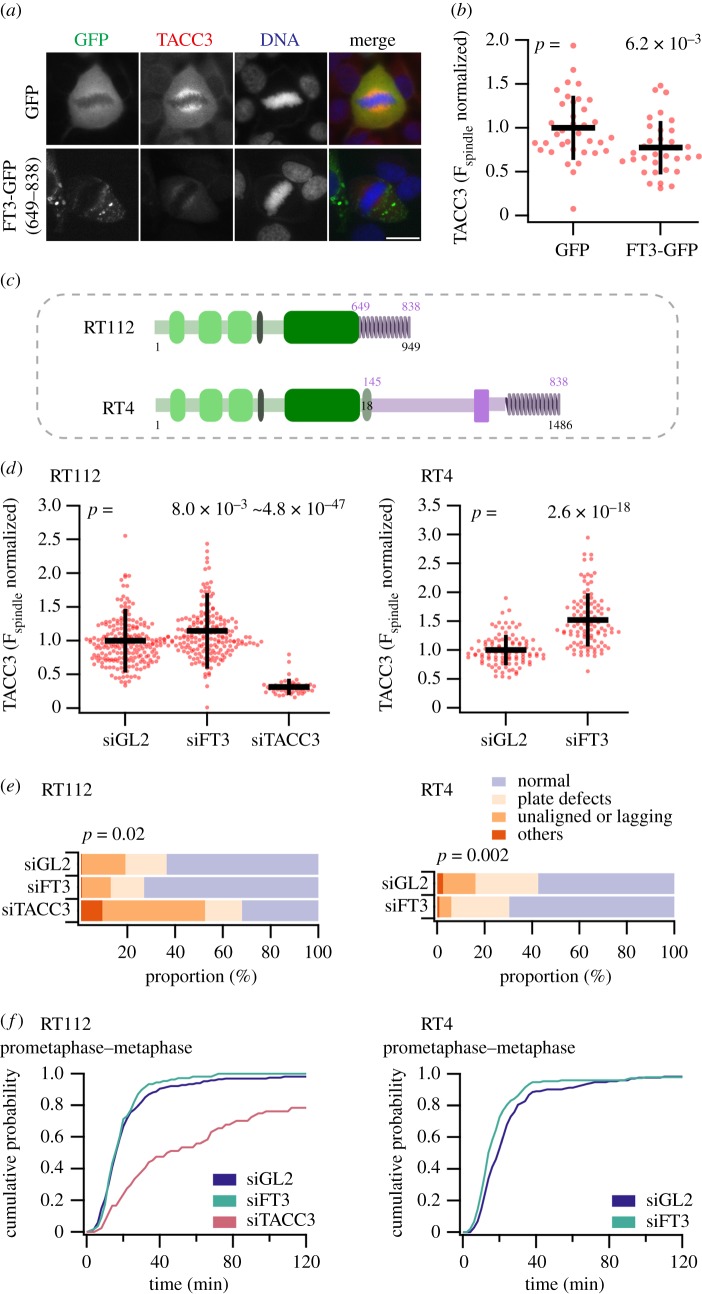
FT3 causes mitotic defects by reducing the level of endogenous TACC3 on the mitotic spindle. (*a*) Representative images of HeLa cells expressing GFP or FT3-GFP(649–838) and stained for TACC3 using an N-terminal antibody. Scale bar, 10 µm. (*b*) Quantification of endogenous TACC3 on mitotic spindles in HeLa cells expressing GFP or FT3-GFP. (*c*) Schematic representation of FGFR3–TACC3 fusions present in RT112 or RT4 bladder cancer cells. (*d*) Quantification of endogenous TACC3 on mitotic spindles in cells transfected with siGL2 or siFT3. Endogenous TACC3 was detected using a TACC3 antibody directed to the N-terminus. Data from RT112 and RT4 cells are shown. TACC3 RNAi was used as a positive control for RT112 experiments. Dots represent individual cells from three independent experiments. Black lines represent mean ± s.d. *p*-Values are shown for ANOVA with Tukey post hoc test (RT112) or Student's *t*-test (RT4). (*e*,*f*) Quantification of mitotic phenotypes (normal and various abnormal phenotypes) observed in RT112 or RT4 cells expressing H2B-mCherry. Cells were transfected with siGL2 or siFT3 (and siTACC3 in the case of RT112 only). Proportions of cells with various mitotic phenotypes are shown (*e*). Cumulative histograms of mitotic progression of all cells (*f*). Prometaphase–metaphase timing is shown for the conditions indicated. RT112: siGL2 *n* = 4, 144 cells; siFT3 *n* = 4, 92 cells, siTACC3 *n* = 4, 84 cells. RT4: siGL2 *n* = 3, 174 cells; siFT3 *n* = 3, 152 cells. The full mitotic progression dataset is shown in the electronic supplementary material, figure S1.

While studying the localization of FT3 in mitotic cells, we noticed that ectopic expression of FT3 caused a reduction in endogenous TACC3 levels on the mitotic spindle (figure 2*a*,*b*). This reduction also caused a partial loss of clathrin and ch-TOG from the spindle (electronic supplementary material, figure S2). In order to investigate the TACC3 removal effect further, we measured the endogenous TACC3 levels on the mitotic spindle in two different FT3-positive bladder cancer cell lines (RT112 and RT4). The FT3 fusions expressed in RT112 and RT4 have similar lengths of FGFR3 but different lengths of TACC3 (figure 2*c*) [3]. The endogenous TACC3 was detected using an antibody against the TACC3 N-terminus, because this part of TACC3 is absent in both FT3 fusions. To test for a role for FT3 in setting the endogenous TACC3 levels on the spindle, we specifically depleted FT3 in RT112 or RT4 cell lines using RNAi and compared this with control RNAi. To do this, we designed siRNAs which specifically targeted the FT3 transcripts in RT112 or RT4 cells (electronic supplementary material, figure S3). Upon knockdown of FT3, the endogenous TACC3 level on the spindle was significantly higher than the control cells in both RT112 and RT4 cells (figure 2*d*).

These results suggest that the presence of FT3 causes a reduction in endogenous TACC3 levels on the mitotic spindle. Previously it has been documented that knockdown of TACC3 causes a delay in mitotic progression together with defects in chromosome segregation [10,17]. In order to test the effect of a reduction in endogenous TACC3 levels during mitosis in the FT3-positive cells, we monitored mitotic progression in these cells. We found that the presence of FT3 causes several mitotic defects including unaligned chromosomes during prometaphase/metaphase and the formation of lagging chromosomes during anaphase (figure 2*e*). Knockdown of FT3 partially rescued these phenotypes. Surprisingly, we did not notice any considerable change in mitotic timing in cells with FT3 versus those with FT3 removed (figure 2*f*). The changes caused by depletion of TACC3 by RNAi in RT112 cells are much more severe suggesting that the removal of TACC3 from spindles in cells expressing FT3 is only partial. These results suggest that the presence of FT3 causes mitotic defects primarily by reducing the level of endogenous TACC3 at the mitotic spindle.

### 2.3. Mild overexpression of TACC3 partially rescues the mitotic phenotypes of FT3-positive RT112 cells

**Figure 3.**
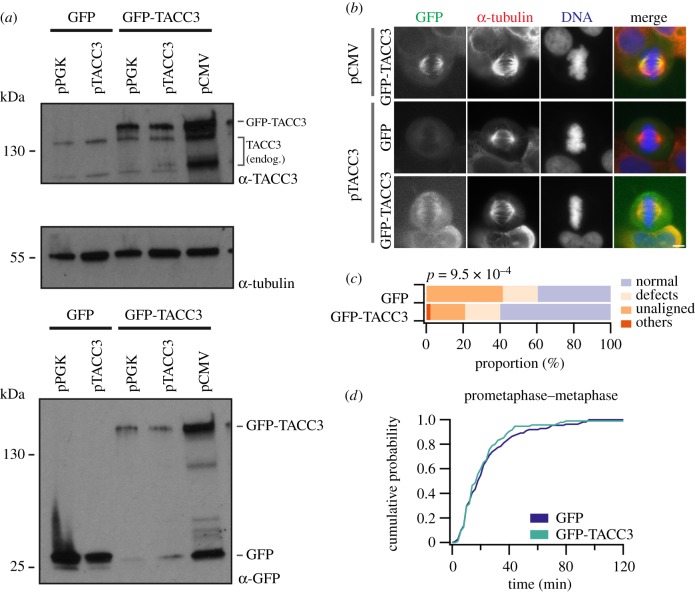
Partial rescue of mitotic defects in RT112 cells upon mild overexpression of TACC3. (*a*) Western blot analysis of TACC3 expression level from different promoters (pCMV versus pTACC3 or pPGK promoter) using α-GFP or α-TACC3 antibodies. α-Tubulin was used as the loading control. pCMV, promoter of cytomegalovirus; pTACC3, promoter of TACC3; pPGK, promoter of phosphoglycerate kinase. (*b*) Representative micrographs of the amount of GFP-TACC3 overexpression under pCMV and pTACC3. Scale bar, 10 µm. (*c*,*d*) RT112 cells co-expressing H2B-mCherry with GFP or GFP-TACC3 under pTACC3, synchronized by a double thymidine block. Movies were started 3 h post-release and imaged every 3 min for the next 12 h. Proportions of cells with various mitotic phenotypes are shown (*c*). Categories are as described for all other figures. Cumulative histograms of mitotic progression of all cells (*d*). Prometaphase–metaphase timing is shown for the conditions indicated. GFP: *n* = 3, 111 cells. GFP-TACC3: *n* = 3, 95 cells. The full mitotic progression dataset is shown in the electronic supplementary material, figure S1.

If the mitotic defects observed in FT3-positive cancer cells are due to reduced TACC3 level on the mitotic spindle, then increasing the level of TACC3 by overexpression in these cells should rescue the defects. Simply overexpressing GFP-TACC3 from a CMV promoter would fail because strong overexpression of TACC3 causes severe mitotic defects in HeLa cells [8]. We therefore expressed GFP-TACC3 at a much lower level using its own promoter to see if we could rescue defects caused by FT3 (figure 3*a*,*b*). Synchronized RT112 cells expressing GFP-TACC3 or GFP together with H2B-mCherry were imaged to monitor mitotic progression. We found that expressing TACC3 at a low level did indeed partially rescue the abnormal mitotic phenotype of FT3-positive RT112 cells (figure 3*c*). Again, we did not notice any significant change in mitotic timing between GFP-TACC3 and GFP positive RT112 cells (figure 3*d*). These results further confirm that mitotic defects in FT3-positive cells are caused by a reduction in the levels of endogenous TACC3 at the mitotic spindle.

### 2.4. Decrease in spindle TACC3 levels is due to a TACC3-specific function of FT3

**Figure 4.**
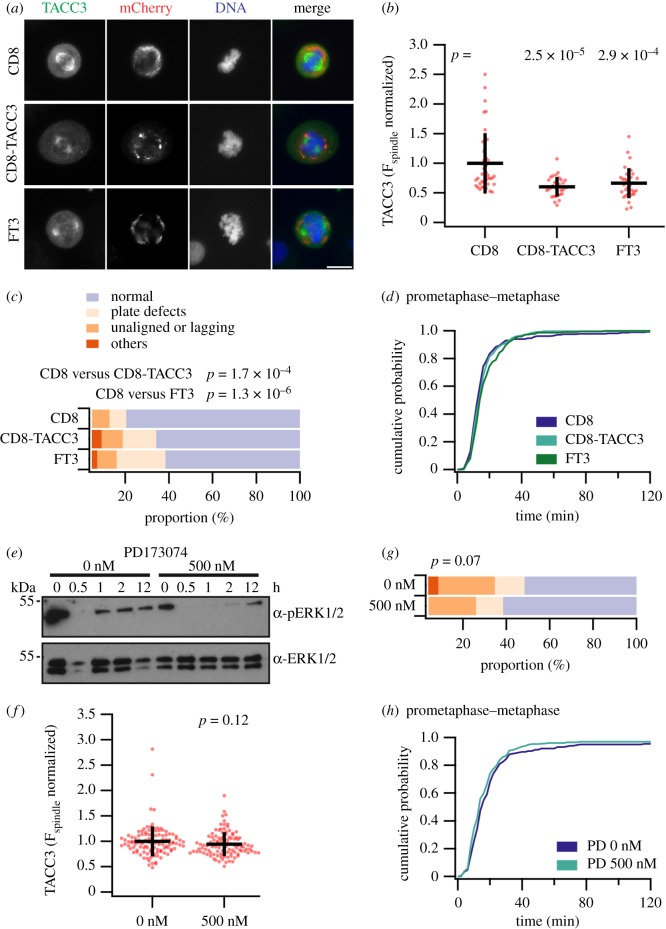
FT3-induced mitotic defects in RT112 cells have a TACC3-specific cause. (*a*) Representative micrographs of cells stained for TACC3 expressing CD8-mCherry, CD8-TACC3-mCherry, or FT3-mCherry. Scale bar, 10 µm. (*b*) Quantitative measurement of TACC3 intensity on mitotic spindle in TERT-B cells transfected with mCherry-tagged CD8, CD8-T3(649–838 aa) and FT3(649–838 aa). Dots represent individual cells from one experiment. Experiment was repeated three times with similar results. Black lines represent mean ± s.d. *p*-Values are shown for ANOVA with Tukey post hoc test. (*c*,*d*) Mitotic phenotype of HeLa cells co-expressing H2B-GFP with the indicated constructs. Cells were synchronized by double thymidine block and 3 h post-release, cells were imaged every 3 min for next 12 h. Proportions of cells with various mitotic phenotypes are shown (*c*). Cumulative histograms of mitotic progression of all cells (*d*). Prometaphase–metaphase timing is shown for the conditions indicated. (*e*) Western blot analysis of phosphoERK signalling in RT112 cells treated with 500 nM PD173074 or DMSO only (0 nM). Whole-cell extracts were prepared at the indicated times, and probed using pERK1/2 and ERK1/2 antibodies. (*f*) Quantification of endogenous TACC3 on mitotic spindles in RT112 cells treated with 500 nM PD173074 or DMSO only (0 nM). Presentation is as in (*a*) *p*-value is from Student's *t*-test. (*g*,*h*) Mitotic phenotype of RT112 cells expressing H2B-mCherry treated with 500 nM PD173074 or DMSO only (0 nM). The proportion of cells with various mitotic phenotypes is shown (*g*). Cumulative histograms of mitotic progression of all cells (*h*). Prometaphase–metaphase timing is shown for the conditions indicated. PD173074 (500 nM): *n* = 3, 239 cells. Control: *n* = 3, 208 cells. The full mitotic progression dataset is shown in electronic supplementary material, figure S1.

How does FT3 decrease TACC3 levels at the mitotic spindle? It could be via a function of the FGFR3 or the TACC3 component of FT3. We tested if the TACC3 component of FT3 was sufficient to reduce endogenous TACC3 levels at the mitotic spindle. To do this, the FGFR3 component of FT3 was replaced with the alpha chain of CD8, a transmembrane protein [23]. CD8-TACC3(649–838) tagged at the C-terminus with mCherry for visualization was expressed in normal TERT-B bladder cells and compared with CD8-mCherry, with no TACC domain, and also with FT3(649–838)-mCherry. We found that in the presence of CD8-TACC3(649–838)-mCherry, the level of endogenous TACC3 on the mitotic spindle was lower compared with CD8-mCherry alone (figure 4*a*). The reduction of TACC3 level due to CD8-TACC3(649–838)-mCherry was comparable to FT3(649–838)-mCherry (figure 4*a*,*b*). We next assessed mitotic progression in cells expressing these constructs together with H2B-GFP in HeLa cells. We found an increase in abnormal mitotic figures in the presence of CD8-TACC3-mCherry compared with CD8-mCherry (figure 4*c*). This increase was similar to expression of FT3 (figure 4*c*). Mitotic progression was unaffected (figure 4*d*). The CD8-TACC3 results strengthen our previous observation that a reduction of TACC3 levels on mitotic spindles increases errors in mitosis. Second, they also suggest a mechanism whereby a TACC domain localized at the plasma membrane can recruit TACC3 away from its site of action on the mitotic spindle. Third, because we could phenocopy the effect of FT3 expression using CD8-TACC3, which lacked an FGFR3 component, it suggests that the mitotic defects are independent of FGFR3 activity.

Although unlikely, we next tested if constitutive signalling from the FGFR3 kinase domain of FT3 can reduce TACC3 levels at the spindle. We measured the endogenous TACC3 level at the mitotic spindle upon inhibition of FGFR3 kinase activity using the small molecule FGFR kinase inhibitor PD173074 [24]. Inhibition of FGFR3 kinase activity was measured by detecting ERK1/2 phosphorylation. FT3 is constitutively phosphorylated in RT112 cells, which leads to increase in FT3 activation and upregulation of ERK1/2 phosphorylation [3]. FT3-driven MAPK signalling in RT112 cells can be inhibited by 500 nM PD173074 (figure 4*e*). We measured the endogenous TACC3 level on the mitotic spindle in the presence of the drug and found no observable change compared with DMSO alone (figure 4*f*). To further investigate whether FGFR3 signalling has any role in the abnormal mitotic phenotype observed in RT112 cells, mitotic progression was monitored in cells treated with 500 nM PD173074 or DMSO alone. We observed only a small increase in the percentage of cells with normal mitosis (figure 4*g*), and no change in mitotic timing (figure 4*h*). This result suggests that FGFR3 signalling has a minor, if any, effect on chromosome segregation in these cells. Whatever its contribution, FGFR3 signalling does not cause removal of TACC3 from the mitotic spindle. Our results clearly indicate that the reduced level of TACC3 at the mitotic spindle is the cause of mitotic problems in FT3-positive cancer cell lines and that this reduction is due to a TACC3-specific function of FT3.

### 2.5. TACC3 region of FT3 can bind and recruit endogenous TACC3

**Figure 5.**
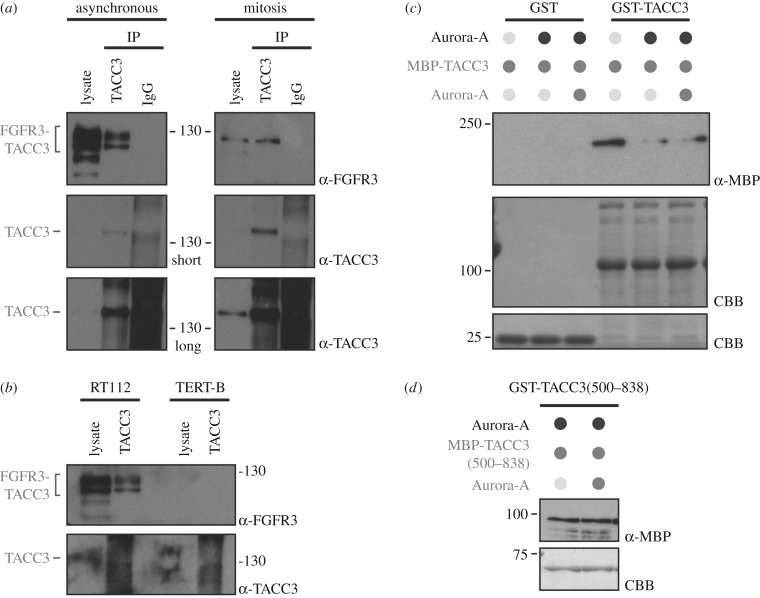
FT3 interacts with endogenous TACC3 via its TACC domain. (*a*) Co-immunoprecipitation of FT3 with endogenous TACC3 in interphase and mitosis. Whole-cell extracts of RT112 asynchronously growing or synchronized in mitosis were subjected for immunoprecipitation using agarose-conjugated TACC3 antibody directed to the N-terminus, followed by western blotting using anti-FGFR3 and anti-TACC3. Long exposure is shown to confirm the presence of TACC3 in the whole-cell extract. (*b*) Co-immunoprecipitation of FT3 with endogenous TACC3 in RT112 versus TERT-B cells, as in (*a*). Note, TERT-B does not have FT3 and was used as a negative control for co-IP of FGFR3 with TACC3. (*c*) Interaction between full-length MBP-TACC3 and GST-TACC3. Western blot to show the amount of MBP-TACC3 bound to GST or GST-TACC3. MBP-TACC3 was detected by anti-MBP antibody and a Coomassie brilliant blue (CBB) gel was run to confirm the presence of GST and GST-TACC3, respectively (same gel is cropped for space reasons). GST or GST-TACC3 was phosphorylated with Aurora-A before incubation with MBP-TACC3 which was unphosphorylated or treated with Aurora-A. (*d*) Interaction between MBP-TACC3(500–838) and GST-TACC3(500–838), as in (*c*). GST-TACC3(500–838) was phosphorylated using Aurora-A kinase before incubation with MBP-TACC3(500–838) which was unphosphorylated or treated with Aurora-A kinase.

Since the TACC3 region of FT3 is primarily involved in removal of endogenous TACC3 from the mitotic spindle, we hypothesized that FT3 must bind endogenous TACC3 to recruit it away from the spindle. If this model is correct, we should be able to detect binding between FT3 and endogenous TACC3. Accordingly, we immunoprecipitated TACC3 using the N-terminal antibody, which would precipitate endogenous TACC3 but not FT3. Using this strategy, we detected endogenous TACC3 selectively binding to FT3 (figure 5*a*). This binding is specific to FT3 because endogenous TACC3 did not co-immunoprecipitate FGFR3 in FT3-negative TERT-B cells (figure 5*b*). The TACC3–FT3 interaction takes place during mitosis in RT112 cells (figure 5*a*).

Since the TACC domain of TACC3 is an obligate dimer and is capable of multimerization [7,19], it seems likely that the interaction between FT3 and endogenous TACC3 occurs via the TACC3 component of FT3 rather than the FGFR3 component. To test this model, we looked to see if differentially tagged TACC3 dimers can bind to each other. Using MBP-TACC3 we could detect binding to GST-TACC3 *in vitro*, but not to GST alone (figure 5*c*). This effect was probably due to the TACC domain which is present in FT3 because we saw a similar interaction when MBP-TACC3(500–838) and GST-TACC3(500–838) proteins were used (figure 5*d*). We also note that Aurora-A phosphorylation of TACC3 is not required for TACC domain multimerization (figure 5*c*,*d*). Overall our data clearly suggest that FT3 is capable of binding endogenous TACC3 and this association takes place via the TACC domain found in FT3.

## 4. Discussion

**Figure 6.**
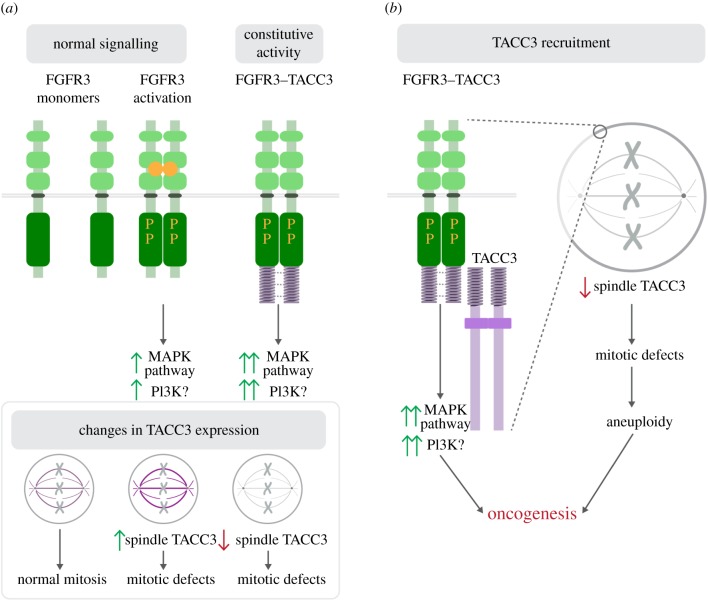
Model to explain how FGFR3–TACC3 contributes to oncogenesis. (*a*) FGFR3 is normally involved in signalling via MAPK and PI3K signalling cascade. Upon fusion with TACC3, FGFR3–TACC3 activates the MAPK pathway constitutively, with possible activation of PI3K. Previous work has described how either a loss or overexpression of TACC3 causes mitotic defects. (*b*) Work in this paper shows that FGFR3–TACC3 binds and recruits TACC3 away from the spindle. In addition to constitutive signalling, cells have defects in mitosis which together are likely to contribute to oncogenesis.

We have studied why FT3 expression in cancer cells results in mitotic defects. We found that FT3 does not localize to the mitotic spindle or centrosomes and is instead found in vesicular structures expected of a transmembrane protein. We showed that the TACC domain of FT3 binds to and recruits endogenous TACC3 from the spindle. This reduction in TACC3 levels on the mitotic spindle is the primary cause of mitotic defects. This mechanism, summarized in figure 6, acts together with aberrant signalling to drive cells towards oncogenesis.

Previous reports suggested that FT3 expression causes severe mitotic defects and delays mitosis in Rat1A cells. It was inferred that this function could be due to its localization at the spindle pole during mitosis [22]. We found that expression of FT3-GFP in HeLa cells induced mitotic defects without being localized to the spindle pole region or mitotic spindle. The localization pattern of FT3-GFP, a transmembrane protein, indicated that it is found in vesicles throughout the cell. This is in agreement with a previous study where FT3 was shown to be localized at the cell surface in RT112 bladder cancer cells [3]. Because FT3 is vesicular and not actually resident at the spindle, an alternative explanation for mitotic defects was required.

Our results suggest that the presence of FT3 induces mitotic defects by removing TACC3 from the mitotic spindle. This is possible due to the TACC3 portion of the fusion. Our evidence for this is threefold. First, FT3 can bind endogenous TACC3 via its TACC domain. This association between FT3 and endogenous TACC3 occurs throughout the cell cycle, however it is only in mitosis where the consequences of the interaction become obvious. Second, we could mimic the effect of FT3 expression by expressing a TACC domain fused to a different transmembrane domain (CD8-TACC3). The degree of mitotic defects is very similar (34% versus 38%) to that observed in FT3-expressing cells. Third, rescue of mitotic defects in FT3-positive bladder cancer cells (RT112 and RT4) was possible either by knocking down FT3 or by low-level overexpression of full-length TACC3. Rescue was incomplete and this is perhaps due to partial FT3 knockdown and/or the variability in TACC3 expression in individual cells upon transient transfection. Together these results suggest that reduced levels of TACC3 on the mitotic spindle is the major reason for mitotic and chromosomal abnormalities observed in FT3-positive cells.

TACC3 is a protein primarily involved in stabilizing kinetochore fibres of the mitotic spindle [25]. These fibres are important for accurate segregation of sister chromatids during mitosis [26–28]. Mammalian cells are sensitive to TACC3 levels because it has been shown that either an increase or a decrease in TACC3 causes mitotic defects and delays in mitosis [8]. Removal of TACC3 by FT3, or indeed by synthetic proteins where a TACC domain is localized away from the spindle, causes mitotic defects. This is likely to be due to a reduction in kinetochore fibre stability [10,17]. Reduced kinetochore fibre stabilization results in a slower progression through mitosis [8,15,17]. The removal of endogenous TACC3 from the spindle by FT3 in this paper did not change the rate of progression through mitosis. This is likely to be because the removal of TACC3 from spindles is not as extensive with FT3 expression as it is with TACC3 RNAi. A direct comparison of the two methods performed here suggests that this is indeed the case.

We also looked at the role of constitutive FGFR3 signalling from FT3 in mitotic defects. We found only a minor change in the number of normal mitoses and no alteration of TACC3 levels at the mitotic spindle. This suggests to us that there is little contribution of altered FGFR3 signalling to mitotic defects observed in FT3-positive cancer cells. Work from other groups has highlighted the role of constitutive FGFR3 signalling in FT3-positive cells [3,29]. The present work suggests that this signalling is likely to influence oncogenesis via mechanisms other than defective chromosome segregation. The importance of signalling versus mitotic mechanisms is evident from work on other oncogenic FGFR3 alterations where a mitotic partner is not evident, e.g. FGFR3(S249C) or FGFR3-BAIAP2L1 [30].

During our work, we noticed that FT3 exists in two forms when cells grow asynchronously. However, during mitosis only the faster migrating form was visible (figure 5*a*). FT3 exists in glycosylated and deglycosylated forms in RT112 cells [31]. It is possible that, during entry into mitosis, FT3 becomes deglycosylated or that the glycosylated form is degraded. The significance of this alteration during mitosis is not known but is interesting for future study.

In this paper, we have shown a TACC3-specific role of FT3 in inducing mitotic defects in bladder cancer cells. The mechanism we describe is general and is likely to translate to other FT3-positive cancer types. This mitotic mechanism occurs simultaneously with constitutive FGFR3–TACC3 signalling. These two mechanisms are likely to work hand-in-hand during oncogenesis, but their relative contributions are uncertain. We note that therapies that inhibit FGFR3 signalling in FT3-positive cancers would not tackle the reduction of TACC3 levels at the mitotic spindle, and mitotic defects will still be present in these cells. The consequences of this for tumour progression are unclear, but development of a therapeutic agent that inhibits signalling and prevents TACC3 removal is most likely to bring FT3-positive cells back to a normal state.

## 5. Material and methods

### 5.1. Cell culture

HeLa cells (HPA/ECACC #93021013) were maintained in Dulbecco's modified Eagle's medium plus 10% fetal bovine serum (FBS) and 100 U ml^−1^ penicillin/streptomycin (pen/strep) at 37°C and 5% CO_2_. RT112 cells (CLS, 300324) were maintained in RPM1 1640 medium supplemented with 2 mM l-glutamine and 10% FBS and 100 U ml^−1^ pen/strep at 37°C and 5% CO_2_. RT4 cells (CLS, 300326-SF) were maintained in EMEM medium supplemented with l-glutamine, NEAA, sodium pyruvate and 10% FBS and 100 U ml^−1^ pen/strep at 37°C and 5% CO_2_. Immortalized normal ureter cells (TERT-B, kind gift from Prof. M. Knowles, Leeds) were maintained in Keratinocyte growth medium kit 2 (Promocell) containing 90 µl of 0.5 M CaCl_2_, puromycin (1 µg ml^−1^) and 100 U ml^−1^ pen/strep at 37°C and 5% CO_2_. For HeLa, plasmid DNA transfection was done by GeneJuice (MerckMillipore, UK), plasmid DNA or siRNA transfection was done either by Lipofectamine 2000 (Life Technologies, UK) in the case of RT112, or by electroporation method (NEON transfection system, Invitrogen, Life Technologies, UK) in the case of RT4 and TERT-B cells, according to the manufacturer's instructions, with the following electroporation parameters (for RT4: pulse voltage 1100 V, pulse width 30 ms and pulse number 1 pulse; for TERT-B: pulse voltage 1200 V, pulse width 30 ms and pulse number 1 pulse).

### 5.2. Molecular biology

To construct FT3(649–838)-GFP, FGFR3 (1–758 aa) and TACC3 (649–838 aa) were each amplified by PCR and ligated to pEGFP-N1 (for GFP) using Gibson assembly. FT3(649–838)-mCherry was constructed by digesting FT3(649–838)-GFP with *Bam*HI and *Age*I and subcloned into pmCherry-N1. To express GFP or GFP-TACC3 under the TACC3 promoter, the endogenous promoter sequence for TACC3 was synthesized by GeneArt, digested with *Ase*I and *Nhe*I and subcloned into either pEGFP-N1 or GFP-TACC3 plasmid. Details of pTACC3 are given in the electronic supplementary material. CD8-TACC3-mCherry was constructed by amplifying TACC3 (649–838 aa) by PCR, digesting with *Bam*HI and *Age*I and cloning into CD8-mCherry, available from previous work [32]. Details of siRNA sequences to specifically knockdown FT3 fusions in RT112 or RT4 cells are given in the electronic supplementary material. Sequences of siRNAs against GL2 (control) and TACC3 are as described previously [33].

### 5.3. Immunofluorescence and microscopy

For measuring endogenous TACC3 levels on the mitotic spindle, cells were transfected with respective plasmid DNA or siRNA, fixed with PTEMF, permeabilized with 0.1% Triton X-100, blocked with 1% BSA, stained with mouse anti-TACC3 and rabbit anti-tubulin and visualized by anti-mouse Alexa488 and anti-rabbit Alexa647 secondary antibodies, respectively. For visualization of TACC3 localization in HeLa, cells were transfected with GFP or FT3-GFP and stained with rabbit anti-TACC3, and for FT3 localization cells were stained with rabbit anti-tubulin or rabbit anti-pericentrin. Clathrin was stained using a similar protocol. For ch-TOG, methanol fixation was used, BSA for blocking and a GFP/A488 was included to boost the GFP signal. All antibodies used in the paper are summarized in the electronic supplementary material.

For live-cell imaging, RT112 or RT4 cells (1 × 10^5^ cells per well, 12-well plate) were transfected with respective siRNAs together with H2B-mCherry, and after 48–67 h cells were imaged on a Nikon Ti epifluorescence microscope with 40× ELWD objective (0.6 NA) and a CoolSnap Myo camera (Photometrics, Tucson, AZ) for 12 h using NIS Elements AR software. H2B-mCherry was imaged once every 3 or 4 min. Cells were kept at 37°C in the presence of CO_2_-free medium. For experiments using GFP-TACC3 rescue or CD8-TACC3 expression, 1 × 10^5^ cells were transfected with H2B-mCherry and GFP-TACC3 or H2B-GFP and CD8-TACC3-mCherry. After 24 h, cells were synchronized at S-phase by double thymidine block, released and mitotic progression imaging was carried out as described above. HeLa cells were used for this experiment because TERT-B cells were difficult to transfect and to image for long periods. For PD173074 experiments, cells (1 × 10^5^ cells) were transfected with H2B-mCherry. Cells were synchronized and imaged as earlier. Cells were kept at 37°C in the presence CO_2_-free medium containing DMSO or PD173074 (500 nM). To avoid light-induced cell damage, intensity and exposure of light were kept to a minimum.

### 5.4. Biochemistry

RT112 cells were seeded into 15 cm dishes at a density of 1 × 10^6^. For mitotic extracts, after 24 h, cells were first synchronized at S-phase with thymidine (2 mM final). After 17 h, cells were washed, released into fresh medium for 6–7 h before being treated with nocodazole (1 µg ml^−1^). Cells were kept in the presence of nocodazole for 16 h, washed and released into fresh medium. After 35–40 min, cells were collected, washed with PBS and lysed in lysis buffer (20 mM Tris–Cl pH 7.5, 137 mM NaCl, 1% NP-40, 5 mM Na orthovanadate, 10 mM β-glycerophosphate, 50 mM NaF, 5 mM tetrasodium pyrophosphate, 100 nM okadaic acid, 15 µg ml^−1^ DNaseI) containing 1 mM PMSF and protease inhibitor cocktail. Cell extract was kept on ice for 30 min before centrifugation at 14 000 r.p.m. for 15 min. Clear supernatant was collected and protein concentration was measured by Bradford assay. For immunoprecipitation, 1 mg of whole-cell extract was precleared using protein A agarose beads pre-equilibrated in wash buffer (described below), supernatant was collected, 10 µg of agarose-conjugated anti-TACC3 (sc22773) was added and kept at 4°C for 2 h with rotation. Beads were collected by centrifugation and washed three times with wash buffer (20 mM Tris–Cl pH 7.5, 137 mM NaCl, 0.1% NP-40 and protease inhibitor cocktail). The beads were resuspended in 1× SDS sample buffer, heated at 95°C for 5 min and run on 7% SDS-PAGE gel. Proteins were transferred to nitrocellulose membrane, blocked in 5% skimmed milk in 1× TBS-Tween 20 and probed with either anti-FGFR3 for detection of FT3 or anti-TACC3 (H300, sc22773) for TACC3 detection. For asynchronous culture, 1 × 10^6^ cells (either RT112 or TERT-B) were seeded onto 15 cm dishes, then after 48 h cell extracts were made and the immunoprecipitation experiment was carried out as described above.

For PD173074-mediated FGFR3 inhibition, 1 × 10^5^ RT112 cells were seeded on 12-well plates. The following day, cells were treated with either DMSO or 500 nM PD173074. Cells were collected, and lysed in RIPA buffer. Total protein concentration was measured by the Bradford method. Whole-cell extract was prepared, and equal amounts of protein taken, heated at 95°C for 5 min and run on a 4–15% gradient gel. Western blotting was carried out using anti-pERK1/2 or anti-ERK1/2.

For measurement of TACC3 levels expressed either from the CMV or TACC3 promoter, RT112 cells were transfected and the following day whole-cell extracts were prepared using RIPA buffer. Equal amounts of protein were taken, heated at 95°C for 5 min and run on a 4–15% gradient gel. Western blotting was carried out using anti-GFP.

For assessment of knockdown, cells were transfected with siRNAs, then after 48–72 h cells were collected, whole-cell extracts made, heated at 95°C for 5 min and run on a 7 or 8% SDS-PAGE, with mouse anti-FGFR3 and mouse anti-tubulin used for detection of FT3 and α-tubulin, respectively.

To test the dimerization/multimerization between TACC3, binding experiments were carried out as previously described [11]. Briefly, GST-TACC3-His6 and MBP-TACC3-His6 proteins were purified using glutathione sepharose 4B and amylose resin, respectively. For *in vitro* interaction studies, equal amounts (50 µg) of GST- and MBP-fused proteins were mixed in reaction buffer I (50 mM Tris–Cl pH 7.5, 150 mM NaCl, 0.1 mM EGTA). The mixture was incubated with a 50% slurry of glutathione sepharose 4B beads (pre-equilibrated in NET-2 buffer (50 mM Tris–Cl, pH 7.5, 150 mM NaCl, 0.5% NP-40 substitute)) and left overnight at 4°C with rotation. Next day, beads were collected by spinning down at 1000*g* for 2 min at 4°C and washed four times with NET-2 buffer. Beads were then resuspended in 30 µl of 1× Laemmli buffer, denatured at 95°C and analysed by western blotting after running on 8% SDS-PAGE. Protein samples were also analysed by staining SDS-PAGE gels with Coomassie brilliant blue. All antibodies used in this study are listed in the electronic supplementary material.

### 5.5. Data analysis

TACC3 intensity on the mitotic spindle was measured by taking the average of four small regions of interest on the spindle and normalizing to the control value. Mitotic progression was monitored by manual annotation of frames where mitotic stage transitions occurred. An automated procedure read in these values and plotted cumulative histograms in IgorPro 7 (Wavemetrics). The code used is available at https://github.com/quantixed/PaperCode. Statistical tests were done in IgorPro using one-way ANOVA with Tukey's post hoc test for more than two experimental groups and Student's *t*-test with Welch's correction for two experimental groups. Binomial data used the χ^2^ test. All figures were made in FIJI, IgorPro and Illustrator.

## Supplementary Material

Supplementary Information

## Data accessibility

Additional data has been uploaded as the electronic supplementary material.

## References

[1] Mertens F, Johansson B, Fioretos T, Mitelman F 2015 The emerging complexity of gene fusions in cancer. Nat. Rev. Cancer 15, 371–381. (doi:10.1038/nrc3947) 25998716 10.1038/nrc3947

[2] Parker BC, Zhang W 2013 Fusion genes in solid tumors: an emerging target for cancer diagnosis and treatment. Chin. J. Cancer 32, 594–603. (doi:10.5732/cjc.013.10178) 24206917 10.5732/cjc.013.10178 PMC3845546

[3] Williams SV, Hurst CD, Knowles MA 2013 Oncogenic FGFR3 gene fusions in bladder cancer. Hum. Mol. Genet. 22, 795–803. (doi:10.1093/hmg/dds486) 23175443 10.1093/hmg/dds486 PMC3554204

[4] Costa R, Carneiro BA, Taxter T, Tavora FA, Kalyan A, Pai SA, Chae YK, Giles FJ 2016 FGFR3-TACC3 fusion in solid tumors: mini review. Oncotarget 7, 55 924–55 938. (doi:10.18632/oncotarget.10482) 10.18632/oncotarget.10482 PMC5342462 27409839

[5] Lemmon MA, Schlessinger J 2010 Cell signaling by receptor tyrosine kinases. Cell 141, 1117–1134. (doi:10.1016/j.cell.2010.06.011) 20602996 10.1016/j.cell.2010.06.011 PMC2914105

[6] Gallo LH, Nelson KN, Meyer AN, Donoghue DJ 2015 Functions of fibroblast growth factor receptors in cancer defined by novel translocations and mutations. Cytokine Growth Factor Rev. 26, 425–449. (doi:10.1016/j.cytogfr.2015.03.003) 26003532 10.1016/j.cytogfr.2015.03.003

[7] Gergely F, Karlsson C, Still I, Cowell J, Kilmartin J, Raff JW 2000 The TACC domain identifies a family of centrosomal proteins that can interact with microtubules. Proc. Natl Acad. Sci. USA 97, 14 352–14 357. (doi:10.1073/pnas.97.26.14352) 10.1073/pnas.97.26.14352 PMC18922 11121038

[8] Nixon FM, Gutierrez-Caballero C, Hood FE, Booth DG, Prior IA, Royle SJ. 2015 The mesh is a network of microtubule connectors that stabilizes individual kinetochore fibers of the mitotic spindle. eLife 4, e07635 (doi:10.7554/eLife.07635) 10.7554/eLife.07635 PMC4495718 26090906

[9] Schneider L, Essmann F, Kletke A, Rio P, Hanenberg H, Wetzel W, Schulze-Osthoff K, Nurnberg B, Piekorz RP 2007 The transforming acidic coiled coil 3 protein is essential for spindle-dependent chromosome alignment and mitotic survival. J. Biol. Chem. 282, 29 273–29 283. (doi:10.1074/jbc.M704151200) 17675670 10.1074/jbc.M704151200

[10] Booth DG, Hood FE, Prior IA, Royle SJ 2011 A TACC3/ch-TOG/clathrin complex stabilises kinetochore fibres by inter-microtubule bridging. EMBO J. 30, 906–919. (doi:10.1038/emboj.2011.15) 21297582 10.1038/emboj.2011.15 PMC3049211

[11] Hood FE, Williams SJ, Burgess SG, Richards MW, Roth D, Straube A, Pfuhl M, Bayliss R, Royle SJ 2013 Coordination of adjacent domains mediates TACC3-ch-TOG-clathrin assembly and mitotic spindle binding. J. Cell Biol. 202, 463–478. (doi:10.1083/jcb.201211127) 23918938 10.1083/jcb.201211127 PMC3734082

[12] LeRoy PJ, Hunter JJ, Hoar KM, Burke KE, Shinde V, Ruan J, Bowman D, Galvin K, Ecsedy JA 2007 Localization of human TACC3 to mitotic spindles is mediated by phosphorylation on Ser558 by Aurora A: a novel pharmacodynamic method for measuring Aurora A activity. Cancer Res. 67, 5362–5370. (doi:10.1158/0008-5472.CAN-07-0122) 17545617 10.1158/0008-5472.CAN-07-0122

[13] Thakur HC et al. 2014 The centrosomal adaptor TACC3 and the microtubule polymerase chTOG interact via defined C-terminal subdomains in an Aurora-A kinase-independent manner. J. Biol. Chem. 289, 74–88. (doi:10.1074/jbc.M113.532333) 24273164 10.1074/jbc.M113.532333 PMC3879581

[14] Fu W, Tao W, Zheng P, Fu J, Bian M, Jiang Q, Clarke PR, Zhang C 2010 Clathrin recruits phosphorylated TACC3 to spindle poles for bipolar spindle assembly and chromosome alignment. J. Cell Sci. 123, 3645–3651. (doi:10.1242/jcs.075911) 20923838 10.1242/jcs.075911

[15] Lin CH, Hu CK, Shih HM 2010 Clathrin heavy chain mediates TACC3 targeting to mitotic spindles to ensure spindle stability. J. Cell Biol. 189, 1097–1105. (doi:10.1083/jcb.200911120) 20566684 10.1083/jcb.200911120 PMC2894451

[16] Schmidt S et al. 2010 The centrosomal protein TACC3 controls paclitaxel sensitivity by modulating a premature senescence program. Oncogene 29, 6184–6192. (doi:10.1038/onc.2010.354) 20729911 10.1038/onc.2010.354

[17] Cheeseman LP, Harry EF, McAinsh AD, Prior IA, Royle SJ 2013 Specific removal of TACC3-ch-TOG-clathrin at metaphase deregulates kinetochore fiber tension. J. Cell Sci. 126, 2102–2113. (doi:10.1242/jcs.124834) 23532825 10.1242/jcs.124834 PMC3666260

[18] Sansregret L, Swanton C 2017 The role of aneuploidy in cancer evolution. Cold Spring Harb. Perspect. Med. 7, a028373 (doi:10.1101/cshperspect.a028373) 28049655 10.1101/cshperspect.a028373 PMC5204330

[19] Burgess SG, Peset I, Joseph N, Cavazza T, Vernos I, Pfuhl M, Gergely F, Bayliss R 2015 Aurora-A-dependent control of TACC3 influences the rate of mitotic spindle assembly. PLoS Genet. 11, e1005345 (doi:10.1371/journal.pgen.1005345) 26134678 10.1371/journal.pgen.1005345 PMC4489650

[20] Guo Y, Scheuermann TH, Partch CL, Tomchick DR, Gardner KH 2015 Coiled-coil coactivators play a structural role mediating interactions in hypoxia-inducible factor heterodimerization. J. Biol. Chem. 290, 7707–7721. (doi:10.1074/jbc.M114.632786) 25627682 10.1074/jbc.M114.632786 PMC4367273

[21] Morris SW, Kirstein MN, Valentine MB, Dittmer KG, Shapiro DN, Saltman DL, Look AT 1994 Fusion of a kinase gene, ALK, to a nucleolar protein gene, NPM, in non-Hodgkin's lymphoma. Science 263, 1281–1284. (doi:10.1126/science.8122112) 8122112 10.1126/science.8122112

[22] Singh D et al. 2012 Transforming fusions of FGFR and TACC genes in human glioblastoma. Science 337, 1231–1235. (doi:10.1126/science.1220834) 22837387 10.1126/science.1220834 PMC3677224

[23] Fielding AB, Willox AK, Okeke E, Royle SJ 2012 Clathrin-mediated endocytosis is inhibited during mitosis. Proc. Natl Acad. Sci. USA 109, 6572–6577. (doi:10.1073/pnas.1117401109) 22493256 10.1073/pnas.1117401109 PMC3340072

[24] Mohammadi M et al. 1998 Crystal structure of an angiogenesis inhibitor bound to the FGF receptor tyrosine kinase domain. EMBO J. 17, 5896–5904. (doi:10.1093/emboj/17.20.5896) 9774334 10.1093/emboj/17.20.5896 PMC1170917

[25] Hood FE, Royle SJ 2011 Pulling it together: The mitotic function of TACC3. Bioarchitecture 1, 105–109. (doi:10.4161/bioa.1.3.16518) 21922039 10.4161/bioa.1.3.16518 PMC3173962

[26] McDonald KL, O'Toole ET, Mastronarde DN, McIntosh JR 1992 Kinetochore microtubules in PTK cells. J. Cell Biol. 118, 369–383. (doi:10.1083/jcb.118.2.369) 1629239 10.1083/jcb.118.2.369 PMC2290046

[27] Rieder CL 2005 Kinetochore fiber formation in animal somatic cells: dueling mechanisms come to a draw. Chromosoma 114, 310–318. (doi:10.1007/s00412-005-0028-2) 16270218 10.1007/s00412-005-0028-2 PMC2570760

[28] Sikirzhytski V et al. 2014 Direct kinetochore–spindle pole connections are not required for chromosome segregation. J. Cell Biol. 206, 231–243. (doi:10.1083/jcb.201401090) 25023516 10.1083/jcb.201401090 PMC4107786

[29] Daly C et al. 2017 FGFR3-TACC3 fusion proteins act as naturally occurring drivers of tumor resistance by functionally substituting for EGFR/ERK signaling. Oncogene 36, 471–481. (doi:10.1038/onc.2016.216) 27345413 10.1038/onc.2016.216 PMC5290037

[30] Nakanishi Y, Akiyama N, Tsukaguchi T, Fujii T, Satoh Y, Ishii N, Aoki M 2015 Mechanism of oncogenic signal activation by the novel fusion kinase FGFR3-BAIAP2L1. Mol. Cancer Ther. 14, 704–712. (doi:10.1158/1535-7163.MCT-14-0927-T) 25589496 10.1158/1535-7163.MCT-14-0927-T

[31] Herrera-Abreu MT, Pearson A, Campbell J, Shnyder SD, Knowles MA, Ashworth A, Turner NC 2013 Parallel RNA interference screens identify EGFR activation as an escape mechanism in FGFR3-mutant cancer. Cancer Discov. 3, 1058–1071. (doi:10.1158/2159-8290.CD-12-0569) 23744832 10.1158/2159-8290.CD-12-0569 PMC3770512

[32] Wood LA, Clarke NI, Sarkar S, Royle SJ In press. Hot-wiring clathrin-mediated endocytosis in human cells. bioRxiv. (doi:10.1101/061986) 10.1083/jcb.201702188 PMC5716275 28954824

[33] Gutiérrez-Caballero C, Burgess SG, Bayliss R, Royle SJ 2015 TACC3-ch-TOG track the growing tips of microtubules independently of clathrin and Aurora-A phosphorylation. Biol. Open 4, 170–179. (doi:10.1242/bio.201410843) 25596274 10.1242/bio.201410843 PMC4365485

