# Supplementary material for: FGFR3–TACC3 cancer gene fusions cause mitotic defects by removal of endogenous TACC3 from the mitotic spindle
Source: Open Biol. 2017 Aug 30;7(8):170080. doi: 10.1098/rsob.170080 (PMC5577446; doi:10.1098/rsob.170080)
Supplement: Supplementary Information [file rsob170080supp1.pdf]

Supplementary Information for Sarkar, Ryan & Royle (2017)

Sequence of TACC3 promoter

```
ATTAATACAGCAGGTTCTGCACCGTGAGCCCCGCCGCGTGGAACTCGTTGGGCGTGAACACGAAGCAC
ACGGCGAACACCAGATAGGCGAGAGTGAAGGTCACCTCGGGGCTGTCCATCGCGCAGCCGCCGGGAAG
CTGTCGAGCTAGGCCCCCGCCCCGGCGCGCGGACGAGGCCGCAGCGCCCAGTCCCGGACCTGTCGGTT
GCGGCGGCCGCCGCCCGGCCGCCCGCGGGGCACTCTAGGACATGGAGTCCCGCCGCCCGGCCGCCCGC
GGGGCACTCTAGGACATGGAGTCCCGCCGCCCGGCCGCCCGCGGGGCACTCTAGGACATGGAGTCCCG
CCGCCCGGCCGCCCGCGGGGCACTCTAGGGCATGGAGTCCCGCCGCTCGGCCGCTCGCGGGGCGCTCT
GGGAGATGCAGTCCCCGTGCGGGTGCGGCCTCTCTTCTCGGCCGGCCCTGGAGGCGGCACCGCACCTG
TGCATTGAGCACAGGTGGGGAAACTTAGGCCTGAGCGAGGCCCTGGCCATGAAGCCCCGCCCAGTAGC
GTCAGTTGCGGAACTCGAAATTCATGGGGCGACTGCGAGCCCGGGGTCTCAGCTTCAGACACCCTGGC
GTGCCCTCCACTCCGGAAACGGAACCGTCGGCGCCCGAGCGGGGGGTGGGGGGGGAGGGAGGGCGGAG
CCGGCGAGGCACAGCTTCCCATTGGACGCCCGCCCTCCTGCGGTCCTGCCCCCGGCCGCGCGCTGATT
GGCCGCGCGAAGGCAACCGTCGGCGTTTGAAACTCCGGCGCGCCGGCGGCCATCAAGGGCTAGAAGCG
CGACGGCGGTAGCAGCTAGGCTTGGCCCCCGGCGTGGAGCAGACGCGGACCCCTCCTTCCTGGCGGCG
GCGGCGCGGGCTCAGAGCCCGGCAACGGGCGGGCGGGCAGGTAGGAGAGCGGCTACACGCTAGC
```

RT4 FT3 (SS49)

CGGGCCUUCUGGCCCAGGUdTdT

RT112 FT3 (SS50)

GUCCACCGACGUAAAGGCGAdTdT

Antibodies

Antibody	Working Dilution	Source
GFP, Mouse	1:1000 WB	Roche
ERK1/2, Rabbit	1:1000 WB	#9102, Cell signaling technology
pERK1/2, Rabbit	1:1000 WB	#4376, Cell signaling technology
FGFR3, Mouse	1:750 WB	B9, sc-13121 Santa Cruz
TACC3, Rabbit	1:1000 WB	H300, sc22773 (1-300 a.a. of hTACC3), Santa Cruz
mCherry, rabbit	1:1000 WB	ab167453, Abcam
Pericentrin, Rabbit	1:5000 IF	ab4448, Abcam
Alpha-Tubulin, rabbit	1:1000 IF	PA5-19489, Thermo
Alpha-Tubulin, mouse	1:5000 WB	DM1A, Sigma
TACC3, mouse	1:1000 IF	ab56595 (1-101 a.a. of hTACC3), Abcam
MBP, mouse 8G1	1:1000 WB	2396, Cell signaling technology
CHC, mouse X22	1:1000 IF	In-house production
Ch-TOG, rabbit	1:5000, IF	34032, Autogen Bioclear
GFP, sheep	1:500 IF	4745-1051, BioRad
Secondary antibodies	1:500 IF	Invitrogen

Figure 1

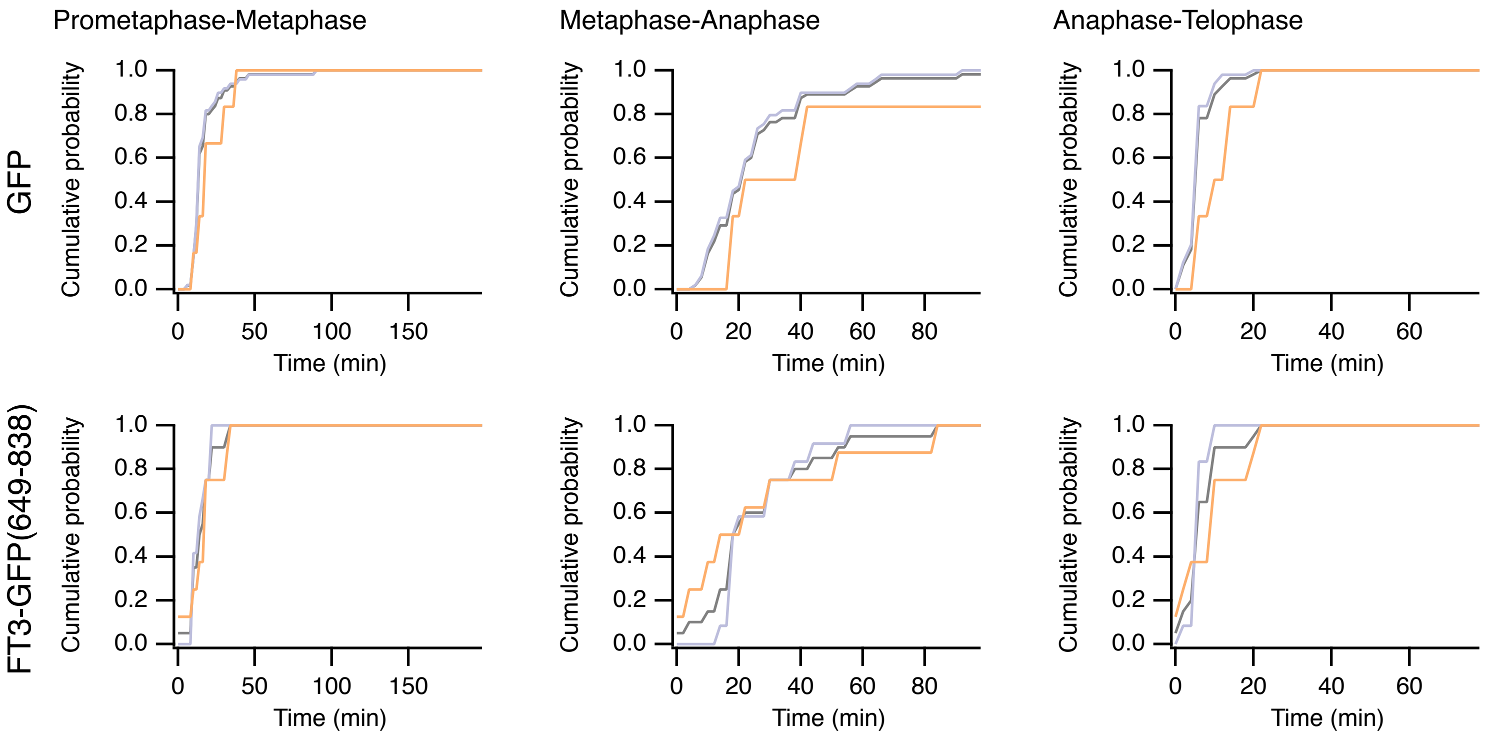

Figure 2F RT4

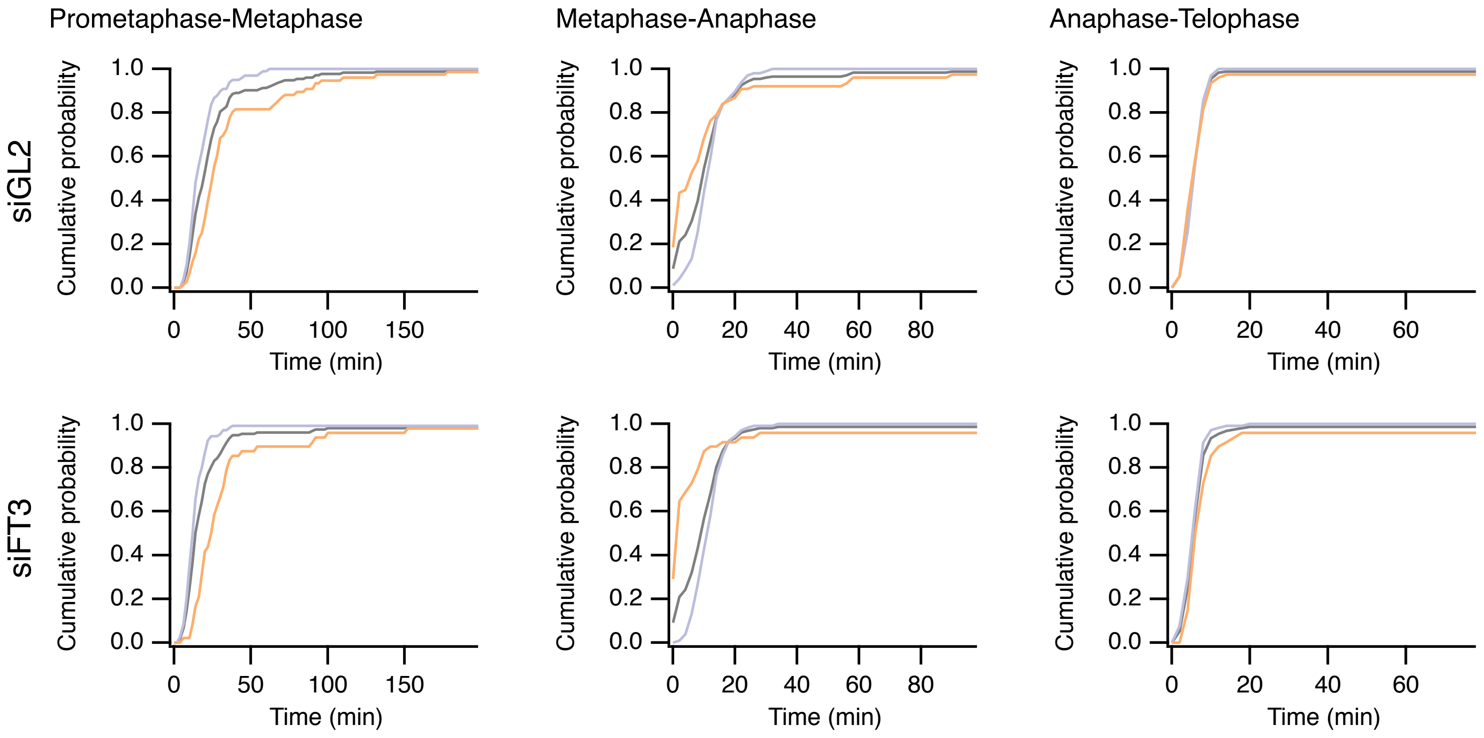

Figure 2F RT112

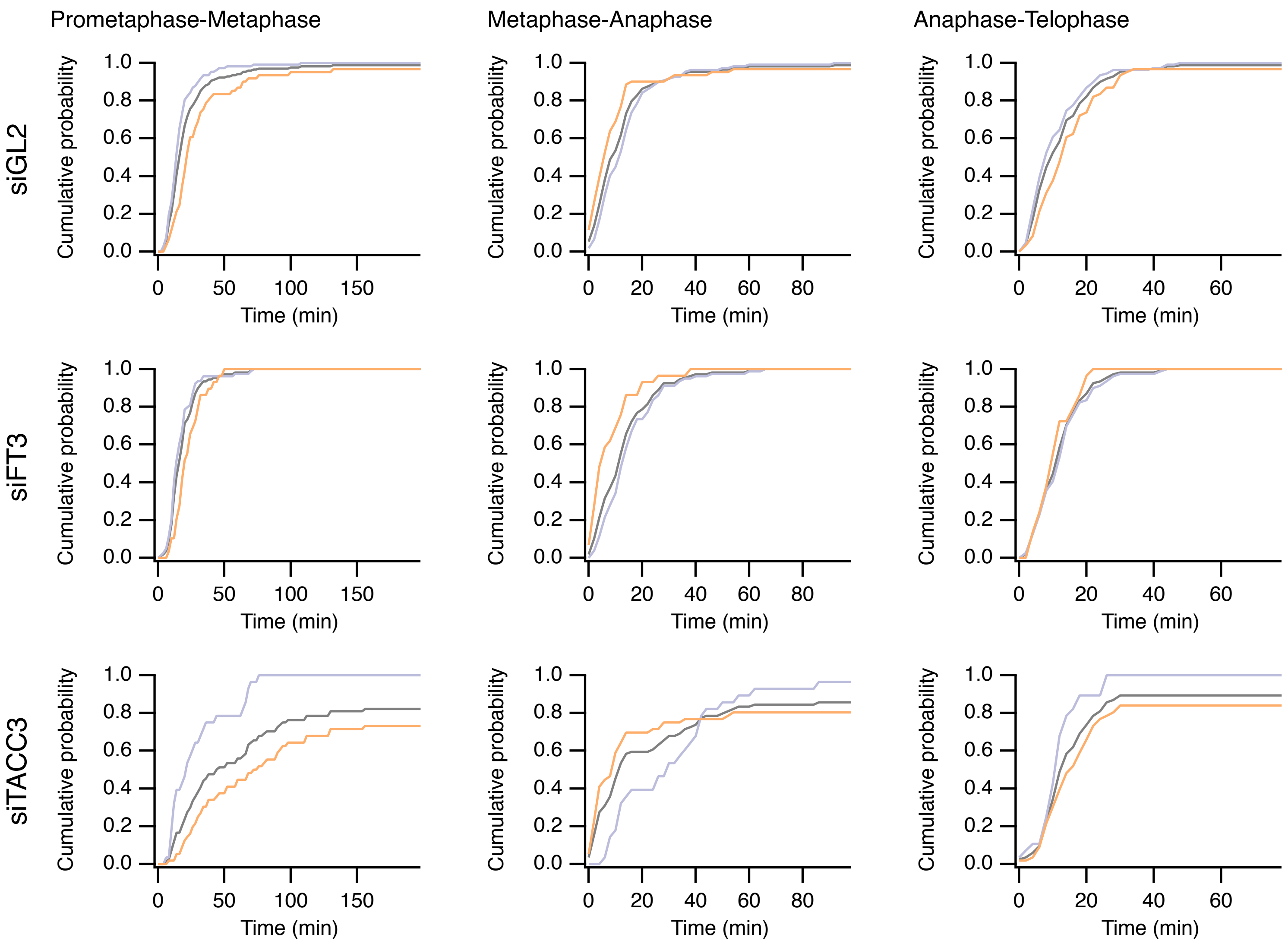

Figure 3D

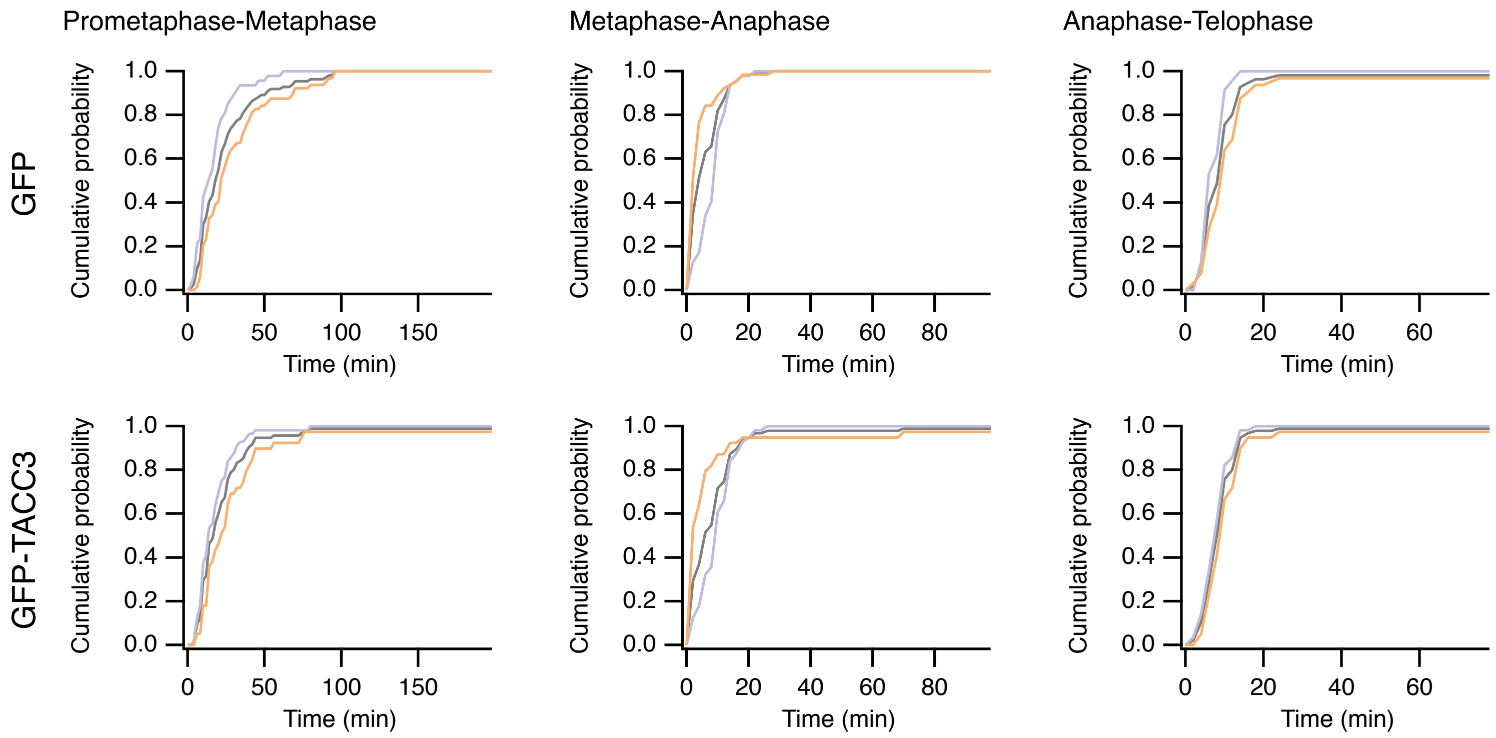

Figure 4D

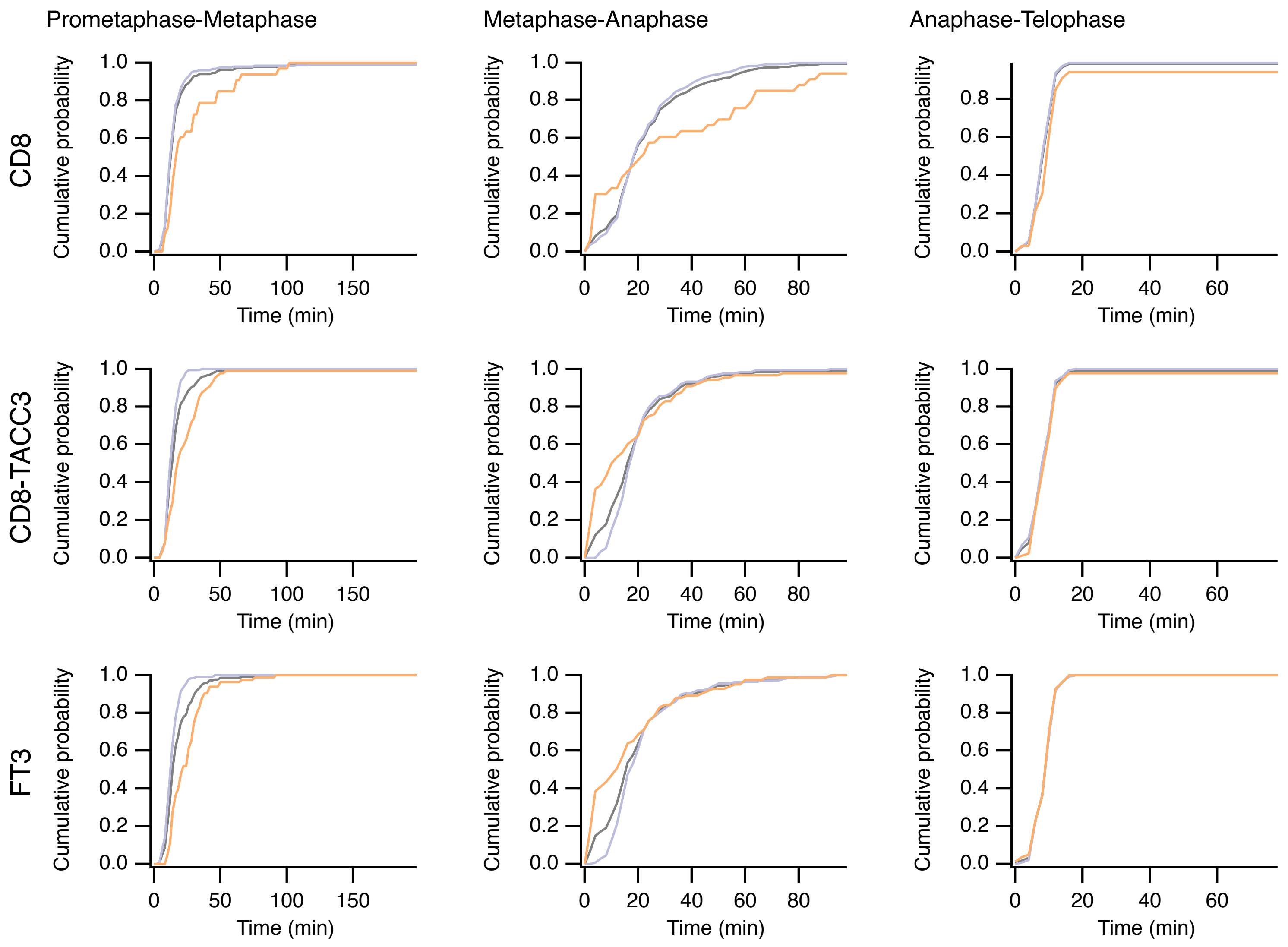

Figure 4H

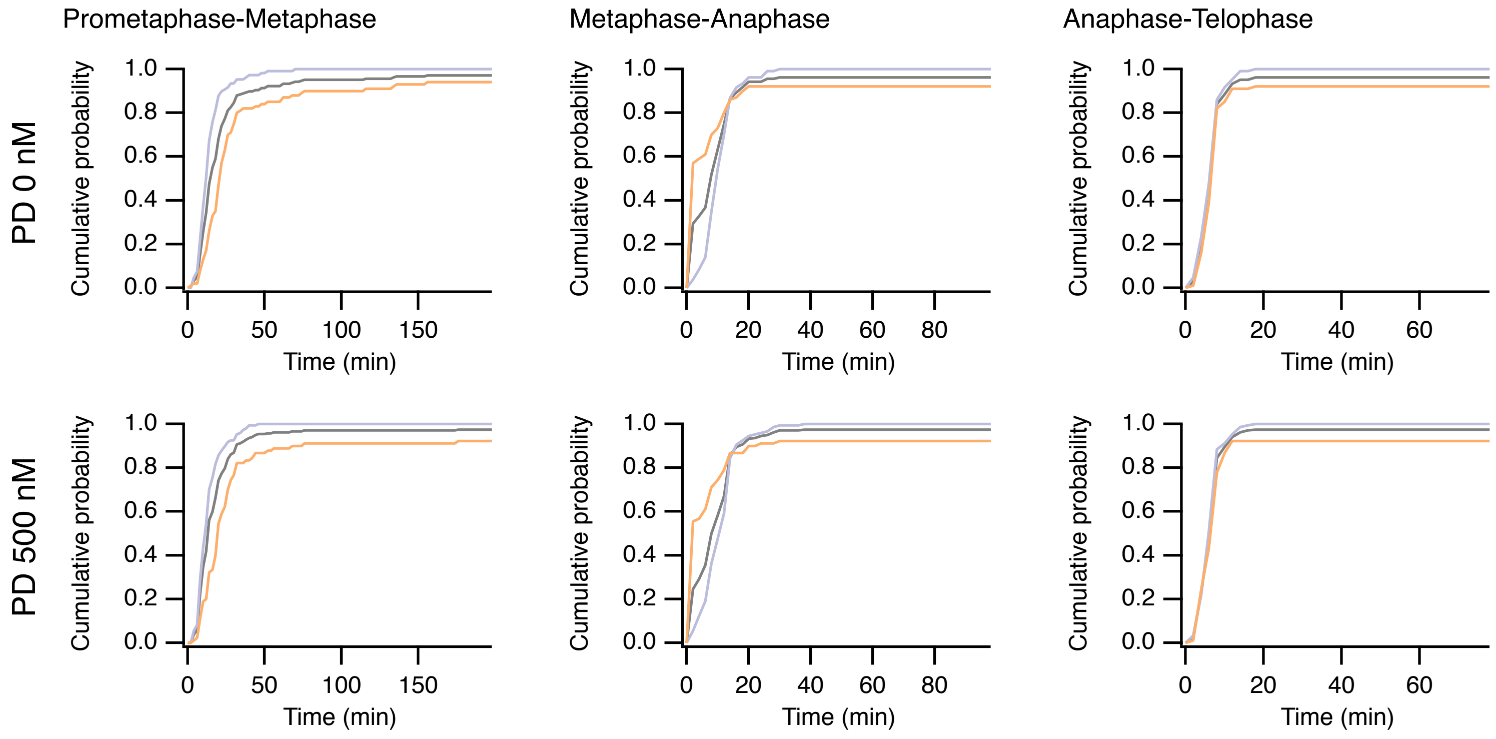

Key: All cells — No defects — Cell with mitotic defects

Supplementary Figure S1. Mitotic progression data for all experiments shown in this paper.

Cumulative histograms of mitotic progression of all cells from the experiments shown in the indicated figures. Prometaphase-Metaphase, Metaphase-Anaphase and Anaphase-Telophase timing is shown (left to right) for the conditions indicated.

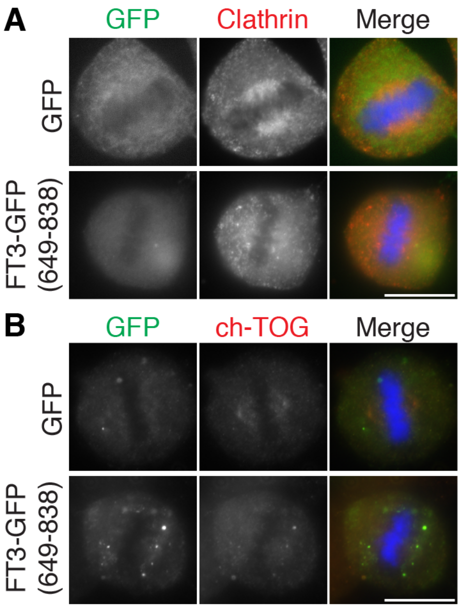

Supplementary Figure S2. Expression of GFP-FT3 in HeLa cells reduces clathrin and ch-TOG on the mitotic spindle.

A. Representative images of HeLa cells expressing GFP or FT3-GFP(649-838) and stained for clathrin (red). Scale bar, 10 μm.

B. Representative images of HeLa cells expressing GFP or FT3-GFP(649-838) and stained for ch-TOG (red). Due to methanol fixation reducing GFP fluorescence, the cells were also stained with anti-GFP/Alexa488 (green). Scale bar, 10 μm.

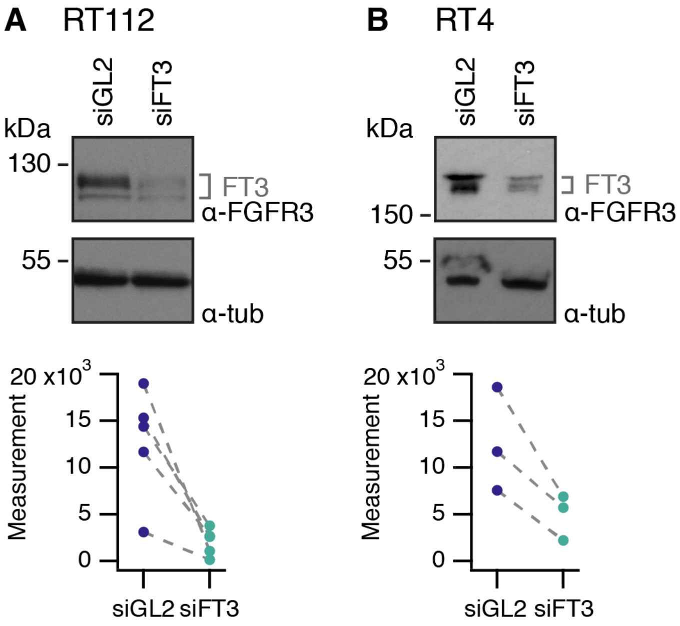

Supplementary Figure S3. FT3 knockdown in RT112 and RT4 cells.

A. FT3 levels in RT112 cells transfected with siGL2 or siFT3. Whole cell lysates were analyzed by immunoblotting using anti-FGFR3 with anti-tubulin as a loading control. Quantification of knockdown is shown for 5 independent experiments.

B. FT3 levels in RT4 cells transfected with siGL2 or siFT3. Whole cell lysates were analyzed by immunoblotting using anti-FGFR3 with anti-tubulin as a loading control. Quantification of knockdown is shown for 3 independent experiments.
